# Ten years on: how far have we come in patient engagement in diagnosis?

**DOI:** 10.1515/dx-2025-0125

**Published:** 2025-09-25

**Authors:** Helen Haskell, Traber Giardina, Io Dolka, Kathryn M. McDonald

**Affiliations:** Mothers Against Medical Error, 155 S. Bull St., Columbia, SC, 29205, USA; Houston Center for Innovation in Quality, Effectiveness & Safety, Michael E. DeBakey Veterans Affairs Medical Center, Houston, TX, USA;; Department of Medicine, Baylor College of Medicine, Houston, TX, USA.; GreyZone Health, Seattle, WA, USA.; Schools of Nursing and Medicine, Johns Hopkins University, Baltimore, MD, USA.

**Keywords:** diagnostic safety, patient engagement, uncertainty in diagnosis, diagnostic equity, patient-reported measures, artificial intelligence in diagnosis

## Abstract

The 2015 National Academy of Sciences, Engineering and Medicine report, Improving Diagnosis in Medicine, is known for its inclusive approach to patients. This paper explores the evolution of research in patient engagement in diagnosis over the past decade, drawing from peer-reviewed literature, policy initiatives, and institutional programs. Major themes include expansion from practical patient aids to co-designed patient reporting systems and patient-reported measures; a focus on diagnostic equity across all populations and conditions; and the emergence of comprehensive multidisciplinary theories framing a “diagnostic ecosystem.” Drivers of change include long-standing frameworks for patient engagement, advances in health information technology, open access to medical records, and regulatory initiatives designed to enhance patient autonomy and enable systematic capture of patient perspectives. Future research in this area should improve patient-reported measures and reporting systems, identify and address diagnostic disparities, and co-create pathways to fully embrace and value the emerging patient voice.

## Introduction

The best-known line in the National Academies of Science, Engineering and Medicine’s (NASEM) report Improving Diagnosis in Health Care is its definition of diagnostic error: “the failure to (a) establish an accurate and timely explanation of the patient’s health problem(s) or (b) communicate that explanation to the patient.” This phrasing was notable for its explicit mention of “the patient”, and inclusion of communication as a criterion for diagnostic success. The NASEM report marked a clear turning point for considering diagnostic quality and safety, which heretofore was focused on the doctor and the healthcare system, not the patient.

The report presented an expansive vision of patient partnership. The report’s first goal, enhancing teamwork and situating patients within diagnostic teams, recommended that patients be educated about the diagnostic process, have access to electronic health records, and be included in efforts to learn from diagnostic errors. Other recommendations acknowledged the importance of communication in areas including improving electronic communication tools and integrating patient communication skills into healthcare curricula [1].

The NASEM report built on earlier work in patient engagement, notably the 2013 overview by McDonald et al., “The patient is in: patient involvement strategies for diagnostic error mitigation.” McDonald et al. highlighted poor communication, poor care coordination, and limited access to health records as patient-reported vulnerabilities within the diagnostic process and proposed a four-part patient-centered research framework: 1) development of a theoretical framework of patient involvement in the diagnostic process; 2) research into factors that encourage or inhibit such involvement; 3) examination of whether and how patient involvement improves diagnostic performance; and 4) evaluation of its impact on outcomes and costs [2].

While there was significant research into patient issues in diagnosis before these two publications, it was largely conducted by physician researchers and reflected the provider perspective. It was the new ethos of patient engagement, a paradigm shift toward patients as active participants, that moved research to meaningfully include patient voices and experiences. Coupled with rising interest in the emerging field of diagnostic improvement, this shift led to an explosion of patient-centered research that continues to evolve. This paper outlines key trends in this research, including major institutional developments and central research themes, along with patient-led efforts and technology advances that impact the field.

## Institutional supporters

Thanks largely to people and organizations associated with the Society to Improve Diagnosis in Medicine (SIDM), diagnosis has gone from being a neglected element of patient safety to being one of its most visible. SIDM’s many achievements are recorded elsewhere in this issue, but the society’s emphasis on patient involvement is worth repeating here. SIDM was conceived as a pluralistic enterprise, and made a point of including patients and patient advocates even before it was formally organized. The 2012 Diagnostic Error in Medicine conference was the first of many SIDM conferences featuring patient speakers throughout the program, with 15 patients included in that particular event [2]. The Patient Engagement Committee (PEC), organized the following year, was one of four board committees representing the four streams (research, education, patient engagement and practice improvement) of the original SIDM strategic plan. Beginning in 2014, free-standing patient summits organized by the PEC brought together researchers and patients to explore diagnostic issues of interest to patients, and members of the PEC have been prominent in research on patient engagement in diagnosis [3].

**Figure 1:**
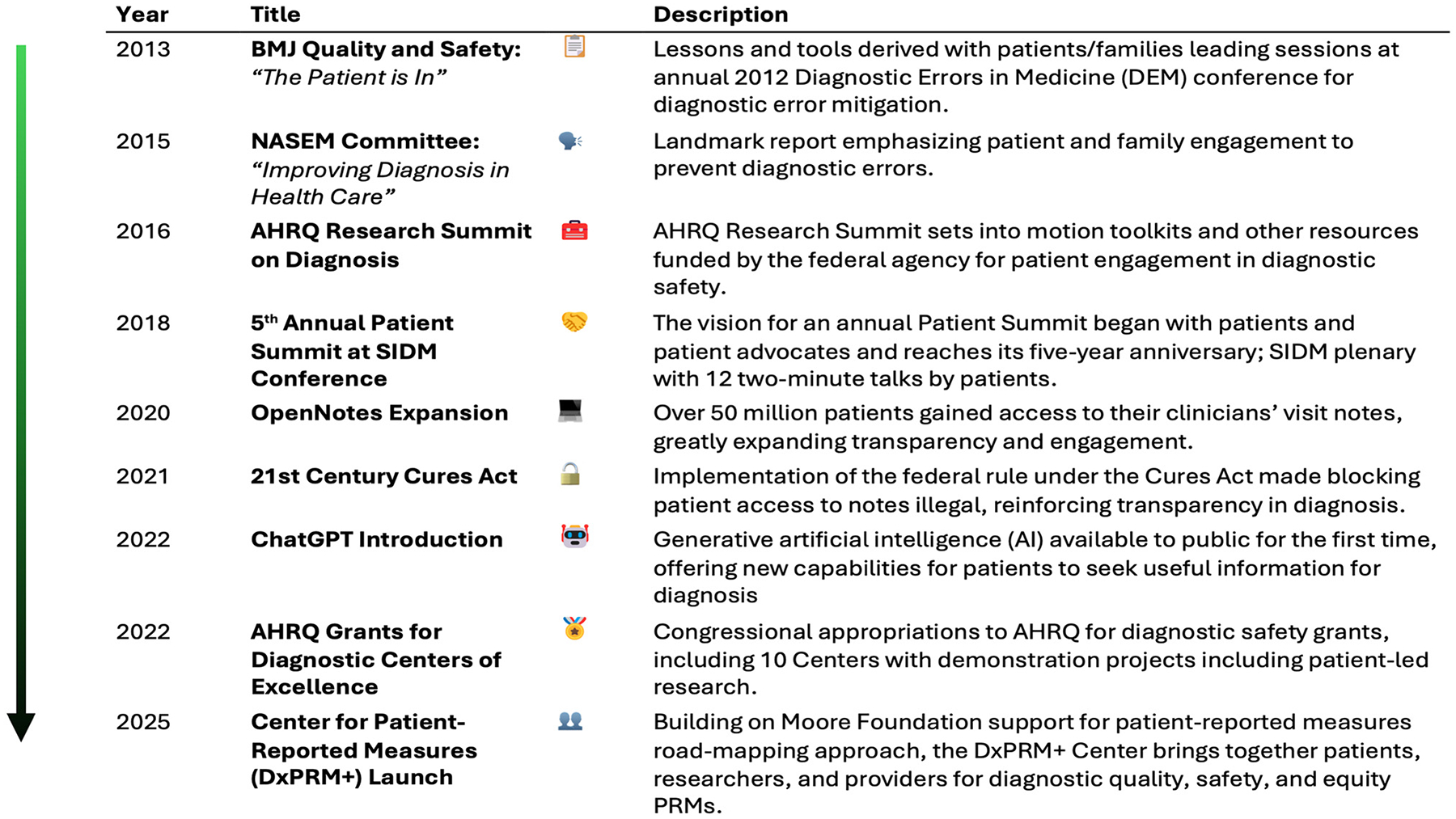
Timeline of significant events in patient engagement in diagnosis, from 2013 publication of first proposed research framework by McDonald et al. to 2025 focus on patient engagement and patient reported measures as essential means of achieving diagnostic quality, safety and equity.

NASEM, in follow-up to the 2015 report, hosts a long-running workshop series and forum on diagnostic excellence, where it has made a point of including patient speakers [4]. The Agency for Healthcare Research and Quality (AHRQ), as the nation’s leading agency for patient safety and quality research, has long supported diagnostic safety work, including SIDM conference funding since 2014. A 2016 AHRQ research summit explored research needs and laid the basis for 10 years of AHRQ diagnostic reports, educational materials, and toolkits, among them Team-STEPPS modules, a Diagnosis Toolkit for diagnosticians and patients, and 28 issue briefs including one authored by patient advocates [5]. In 2022, 10 centers for diagnostic excellence were approved by Congress and administered by AHRQ. Almost all the centers for diagnostic excellence incorporate a patient engagement component into their research [6]. One of them, the Patient-Partnered Center for Diagnostic Excellence, works with a large advisory group exclusively on patient issues [7]. The Leapfrog Group healthcare rating organization, in a multi-year project, has curated and promulgated best practices in diagnosis for hospitals, including diagnosis-centered patient advisory councils, while the Coordinating Center for Diagnostic Excellence (CoDEx), founded in 2024, carries diagnostic improvement forward with learning and engagement programs to reach the wider diagnostic community [8, 9]. This and much other research in both diagnosis and patient engagement, funded primarily by the Gordon and Betty Moore Foundation, has laid the groundwork for fundamental changes to the way that healthcare providers and patients expect to interact (Figure 1).

## Uncertainty and the problem of dismissal

**Figure 2:**
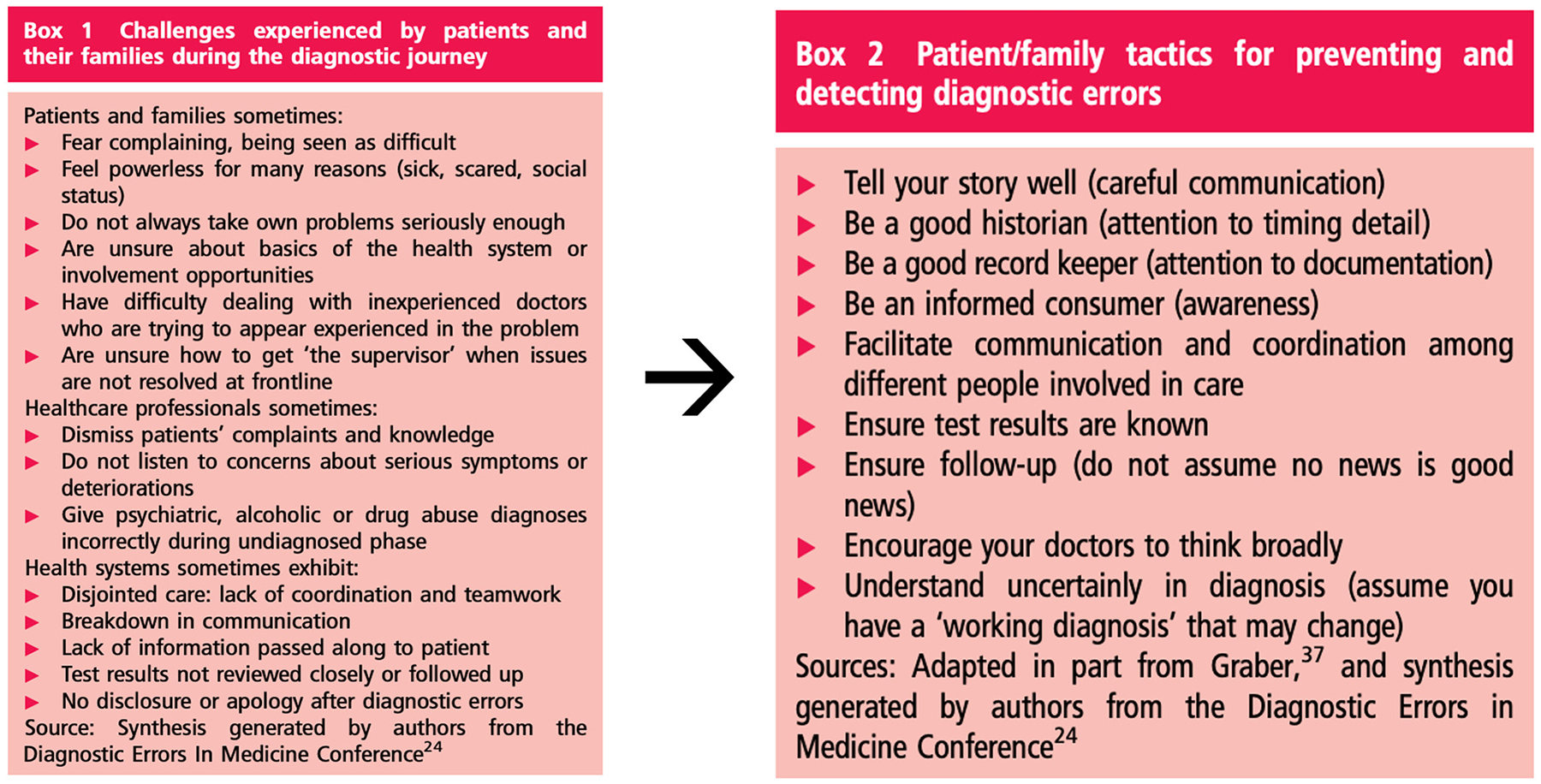
Diagnostic challenges cited by patients and proposed tactics for addressing them, 2013. Reproduced from McDonald KM, Bryce CL, Graber ML. The patient is in: patient involvement strategies for diagnostic error mitigation, BMJ Qual Saf. 2013 Oct;22 Suppl 2(Suppl 2):ii33-ii39 with permission from BMJ Publishing Group Ltd.

Early efforts on patient engagement in diagnosis focused primarily on patient storytelling and patient-provider communication. “The patient is in” chronicled patient suggestions (Figure 2), while the PEC created the SIDM patient toolkit for diagnosis and featured educational materials and communication advice in its early patient summits. A later grant from the Patient-Centered Outcomes Research Institute (PCORI) highlighted using the NASEM process diagram to help patients interpret their stories [10]. The often un-stated motivation for these efforts was and remains the challenge inherent in the uncertainty of diagnosis, a vexing topic for diagnosticians and patients alike. Literature aimed at providers includes recommendations for shared decision-making in diagnosis [11], guiding principles for communicating uncertainty [12–14] and tools for dealing with uncertain diagnoses [15]. A 2021 review by Meyer et al. mapped different areas of uncertainty to the steps of the NASEM diagnostic process and offered recommendations for diagnosticians to manage communication of uncertainty [16]. Australian researchers Dahm and Crock have published on communication, uncertainty and understanding in diagnosis from a linguistic point of view and the importance of conveying to patients an understanding of the uncertainty and complexity of diagnosis [17].

Patient researchers typically take an inclusive view of uncertainty and its potential influence on diagnostic reasoning, often based on patient reports of provider stereotyping and dismissal of symptoms. The list of potentially affected populations is long, including women with pelvic pain, patients with medically unexplained symptoms, and people who have disabilities, obesity, mental illness, or come from racial/ethnic or socioeconomic minorities. These topics occupy a large place in the writing on patient engagement, not only in the medical literature but in the popular press and even on social media [18, 19]. Endometriosis, especially, has gained attention in recent years as a relatively common but debilitating condition whose symptoms are frequently dismissed. Bontempo, a patient-provider communication researcher, has used endometriosis as a window onto what she calls “invalidated symptoms,” that is, practitioners not listening to or believing patients and the long-term consequences this can have [20].

Dismissal and disrespect are recurring themes in patient interviews and surveys. Perceived attitudes of dismissal and disrespect have been correlated with error, especially diagnostic error [21]. Uncertainty, in general, in spite of its high priority in studies of patient engagement in diagnosis, remains a fraught issue for clinicians, who often have an additional layer of misgiving as to how patients will receive an admission of uncertainty [16]. When questioned, however, many patients say they prefer honesty over certainty and welcome a discussion of uncertainty as long as it is accompanied by a plan to address the evolving diagnosis [20].

## Equity in the context of diagnosis

Health equity, the concept that everyone should have a fair and just opportunity to attain their highest level of health, regardless of social, economic, or geographic factors, has far to go in the United States. This includes equity in diagnosis, where issues such as stereotyping or lack of cultural awareness on the part of the provider, and educational or linguistic limitations on the part of the patient, can affect communication and cloud thinking. Equity was a later addition to the study of patient engagement, but as researchers began to grasp its significance and complexity, it became a major focus and now underlies a significant portion of research into patient engagement in diagnosis. Overall, diagnostic equity remains a difficult concept to pin down, in part because of its multiple overlays of social, economic, educational, and medical factors [22]. Approaches have included studies aiming to quantify the nature and distribution of diagnostic disparities [23, 24] and looking at gaps and potential solutions in the healthcare system [25]. Giardina et al. suggest addressing these issues by raising cultural awareness among clinicians, reaching out to communities as partners in the healthcare endeavor, and using technologies like telehealth to make healthcare more accessible [26].

## Open records and patient reporting

The quest for access to information has been a driving force in the patient movement. Patients have continuously sought access to their medical records, information on their conditions, and clarity on the inner workings of the medical system, including the diagnostic process. Twenty-first century technological advances, including patient portals and accessible electronic medical records, have significantly changed both the availability of information and the ability to analyze it. A pivotal advance was the OpenNotes movement, founded in 2010 to pilot the then-controversial concept of doctors sharing their outpatient visit notes with patients [27]. A 2020 survey found that one in five OpenNotes users reported mistakes in their visit notes, including diagnostic errors [28]. The OpenNotes group subsequently created a primary care pre-visit form allowing patients to upload their own versions of the medical history and problem list and asking providers to incorporate them into their visit notes. Both patients and providers responded positively to this intervention [29]. Using the findings of their OpenNotes surveys along with a large national medical error survey, Bell et al. worked with patients to develop a comprehensive framework for identifying diagnostic breakdowns in ambulatory care, the Patient-Reported Diagnostic Breakdown (PRDB) framework [30]. Using information from the PRDB framework and pre-visit surveys, they tested patient responses for diagnostic breakdowns that might not otherwise be known to clinicians, and created and tested OurDX, an online pre-visit questionnaire designed to set the patient’s agenda and also detect any diagnostic breakdowns or concerns the patient might have experienced [31, 32].

Beyond records, patient reports offer a direct view into diagnostic experiences. Giardina et al. analyzed 184 patient-reported diagnostic encounters, revealing 224 instances of unprofessional provider behavior that patients believed led to harm [33]. The same research group reviewed patient complaints and associated medical records and identified patterns in diagnostic errors, noting that patients often described diagnostic concerns without using the word diagnosis [34]. Similarly, Smith et al. found reports of diagnostic breakdowns in free-text comments on patient experience surveys from urgent care centers [35]. Baker et al. found correlations between structured patient experience survey responses and free text responses regarding diagnostic breakdowns, suggesting potential for streamlined methodologies to trigger deeper investigation [36]. Fisher et al. noted greater success with interview probes about breakdowns in care with hospitalized patients compared to web-based methods to acquire these reports [37].

In another approach, Giardina and colleagues developed the Safer DX Patient Instrument, an 11-question survey designed for patient portal integration, to enable collection of patient-reported diagnostic concerns [38]. Among those who identified diagnostic concerns, provider thoroughness and trust issues emerged as major factors. A subsequent chart review study of these cases found little agreement between patient and provider interpretations of the same encounter, suggesting that without the patient perspective, important diagnostic safety concerns may go undetected [39].

Gleason and colleagues developed and validated PRIME-ED, a measure of patient-reported diagnostic quality in emergency and urgent care. Tested with over 1,000 patients, it can be used in follow-up procedures to capture patient perceptions of the diagnostic process and flag cases for review. Unlike tools focused solely on detecting diagnostic errors, PRIME-ED measures whether patients and families left the medical encounter with the information they needed [40]. Dukhanin and colleagues analyzed 982 free-text PRIME-ED responses, identifying several types of “patient reasoning” used to assess diagnostic accuracy, including evidence corroboration, assessing relevant perspectives, and avoiding unsupported assumptions [41].

These studies have coincided with and built upon the work of the National Quality Forum (NQF), which looks at overall approaches to measuring diagnostic performance and developing patient-reported measures (PRMs) for diagnostic excellence. NQF reports in 2017 and 2020 recommended the use of PRMs to measure and promote patient inclusion in the diagnostic process [42, 43]. An ongoing NQF initiative, Advancing Measurement of Diagnostic Excellence, focuses on overcoming major barriers to diagnostic measurement, with emphasis on PRMs, diagnostic equity, and artificial intelligence [44].

## Scaling the challenge

Collecting data on diagnostic safety has long been a challenge, particularly when trying to capture patient perspectives. However, researchers have made strides to elicit patient narratives at scale, combining nationally representative surveys with qualitative accounts of lived experiences [45]. In a two-part AHRQ diagnostic safety issue brief, Schlesinger et al. introduced the application of these methods to diagnosis and emphasized that only patients and families know certain parts of the healthcare experience, insights that may be invisible to clinicians and healthcare systems. Their approach distinguishes between time-limited safety problems that can be captured via surveys within a specific setting and those involving multiple settings or long-term harms, which can be better assessed with large-scale population surveys [46, 47].

A national survey conducted with the NORC AmeriSpeak panel highlights this potential. Of the 3,995 respondents, 1,506 reported experiencing at least one diagnostic “mistake or problem” in the previous four years. Findings showed significant disparities: diagnostic incidents were most prevalent among younger individuals, low-income households, heterosexual women, gender-diverse individuals, multiracial respondents, and people unable to work due to disability. Surprisingly, older adults and most racial and ethnic minority groups did not report higher rates of diagnostic problems or mistakes, suggesting that diagnostic inequity may manifest in ways not traditionally measured. This survey serves as proof of concept that carefully designed population surveys can identify patterns in diagnostic experience often missed in smaller-scale work [23].

Transforming patient-reported narratives into meaningful measures of diagnostic quality requires more than better survey design – it demands a rethinking of whose knowledge counts, how equity is operationalized, and what systems need to change. Krishnan et al. proposed a socioecological model with visual elements and descriptive examples that link below-the-surface and atmospheric conditions that together change the terrain through which a patient’s journey to a diagnosis might play out with great difficulty or much more smoothly, depending on the terrain features (e.g., craggy mountains vs. open pathways) [22]. This perspective complements that of McDonald et al., which offers a structured yet flexible guide to patient-reported measures (PRMs) that center patient experience and equity from the ground up. Their approach moves away from clinician-dominated definitions of diagnostic excellence, framing PRMs as tools to expose gaps in diagnostic continuity, communication, and follow-up, especially those that disproportionately affect vulnerable populations. These roadmaps articulate how PRMs should be co-developed, scaled, and used to drive systemic accountability over the next decade [48]. This approach builds on a scoping review of PRM domains, and pairs specific measurement opportunities with cases of diagnostic error and diagnostic excellence [49, 50].

Further building on this foundation, Srivarathan & Giardina introduce a “landscape of diagnosis” model that situates diagnostic safety within patients’ lived realities within their communities. By integrating patient safety science with community engagement strategies from public health, they reframe diagnostic safety as a shared responsibility. They argue that diagnostic equity requires healthcare systems to partner with the community-based organizations, cultural intermediaries, and social networks that patients navigate long before and after clinical encounters. Their strategies, such as embedding community health workers and fostering neighborhood-level diagnostic partnerships, shift diagnostic safety from system-centric metrics to a community-driven practice [51].

## Patient autonomy

As the patient engagement literature reflects, patients are now an active part of the healthcare system. They routinely serve on patient advisory councils, as independent advisors, as partners in research, and as policy experts. An even more profound influence on the patient role in healthcare, though less frequently a topic of diagnostic research, is the evolving behavior of the public. Patients are embracing a more active role in the diagnostic process, with younger generations such as Millennials and GenZ demanding a more patient-centric healthcare experience and having a preference for self-service options [52]. Publicly available information and tools on the Internet have brought sweeping changes, fundamentally altering how patients research, monitor, and manage their health conditions. More than half of Americans now research health information online, gaining access to medical knowledge that was once the exclusive domain of healthcare providers. Although usage varies across racial and ethnic groups, in a 2022 survey at least 45 % of adults in every group said they used the Internet for medical purposes [53].

One of the first types of tools developed, online symptom checkers such as WebMD and Isabel, allows laypeople to self-triage and self-diagnose, offering the average patient an opportunity for empowerment and agency by democratizing access to medical knowledge and helping individuals become more informed partners in their healthcare journeys [54, 55]. The diagnostic accuracy of such tools varies among different outlets and studies. A systematic review by Riboli-Sasco et al. showed that when patients use these diagnostic tools, accuracy rates are overall lower than general practitioners achieve. Additionally, on average, online symptom checkers perform better on diagnosing common conditions than on rare diseases [56].

**Figure 3:**
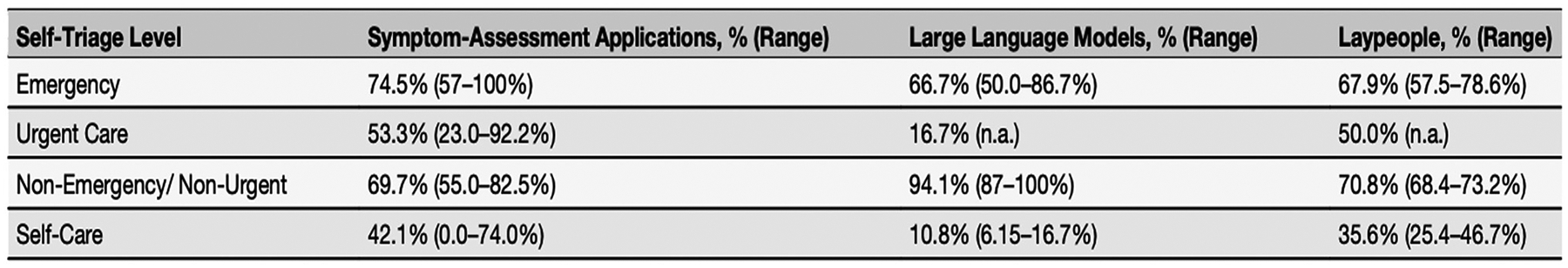
Comparison of reported diagnostic accuracy of online symptom checkers, individual patients, and artificial intelligence chatbots/large language models. Reproduced from Kopka M, von Kalckreuth N, Feufel MA, accuracy of online symptom assessment applications, large language models, and laypeople for self–triage decisions, *npj Digit*. *Med*. 8, 178 (2025).

The emergence of artificial intelligence (AI) technologies, including Large Language Models (LLM), has enhanced patient empowerment and agency through access to clinical reasoning and comprehensive responses to complex medical inquiries, successfully translating medical expertise into comprehensible information for non-expert patients [57]. Patients use AI in increasing numbers, with technology capabilities increasing at a rapid rate. A recent literature review looked at the accuracy of recommendations regarding whether and where medical care should be sought (self-triage accuracy), comparing online symptom checkers to LLMs and laypeople. Online symptom checkers’ triage accuracy had a high variability range from 26 to 88 %. They were the most accurate in emergency cases (74.5 %). LLM triage accuracy was the highest, with a low variability range from 58 to 70 % and was the most accurate in non-emergency/non-urgent cases (94.1 %). Laypeople’s triage accuracy was the lowest and had a low variability range from 47 to 62 % [58] (Figure 3).

Generative AI capabilities are widely regarded as potentially revolutionary for healthcare autonomy, even though – at this time – inherent limitations and error risks are a significant issue. Stories abound of patients using AI to uncover elusive diagnoses that had previously confounded their doctors, or being given life-saving warnings by AI after being sent home from the emergency room [59]. In a small study, individuals with spinal cord disease or injury rated ChatGPT as comparable to medical professionals in its assessment and management recommendations for urinary symptoms [60]. In addition, one study indicated that patients rate AI higher in compassion than doctors [61]. On the other hand, LLMs are frequently found to exhibit racial and gender biases, as well as not only inheriting existing cognitive biases, but potentially intensifying them. Increasing reliance on AI-generated medical guidance, if not undertaken with caution, has the potential to contribute to diagnostic errors and pose serious risks to individuals seeking care [62]. Nevertheless, for all its shortcomings, AI can provide patients with information and resources that may bolster their sense of agency and ability to fully participate in healthcare decisions and the diagnostic process.

Other uses for AI receive mixed reviews from patients. The Patient-Partnered Center for Diagnostic Excellence solicited patient perspectives in a focus group on the use of AI in various areas of medicine (ambient scribe, radiology review, decision support, portal communication, and a virtual human making and transmitting the diagnosis). They found reasonable acceptance, though not wild enthusiasm, for all interventions except the virtual human. Concerns about AI use included reliability, accuracy, privacy, and opportunity for consent [63]. At the same time, telemedicine’s widespread availability and the consistent patient willingness and desire to use it, especially among those with complex health care needs and for those wishing to include others in the visit [64], remove geographical and time barriers to healthcare access, potentially enabling earlier medical consultation for conditions that might otherwise face diagnostic delays due to limited provider availability. Second opinions are also more readily available from reputable sources online, such as Mayo and Cleveland Clinics and Johns Hopkins. Even more transformative is the fact that more than 65 % of patients now use patient portals to access their medical records and communicate with providers, creating a conduit for greater interaction and improved health literacy [65].

Throughout the 2010s, social media, particularly Twitter, was a rallying point for patients as well as medical communities. This has been significantly affected by the conversion of Twitter to X, and a comparable home for communities of interest has not really been found. While support groups on Facebook and elsewhere continue to have large followings, many patient stories of diagnosis and misdiagnosis have moved to TikTok and YouTube [66]. Individual patient advocates, like diagnosis advocate Helene Epstein, also blog on Substack [67].

Patient advocacy groups have been active since the earliest days of the patient safety movement, notably Ilene Corina’s PULSE Center for Patient Safety Education and Advocacy [68]. More recently, professional patient advocacy has emerged as a growing healthcare specialty, primarily composed of nurses and other healthcare professionals who have transitioned from conventional practice settings to focus specifically on patient navigation and care coordination. This field has developed its own credentialing system through the Patient Advocate Certification Board (PACB), which offers the Board Certified Patient Advocate (BCPA) credential, though certification remains voluntary rather than universally mandated [69]. This field has largely replaced the independent consumer advocates and self-help manuals of earlier years.

Several professional advocates focus on assisting patients with diagnostic care advocacy or navigation in particular. A novel patient advocacy model (GreyZone Health) has been developed to address challenges faced by individuals with medically unexplained symptoms and complex diagnostic presentations. This evidence-informed approach integrates personal experience with diagnostic odysseys and published research on diagnostic error sources. The methodology centers on four core interventions: meticulous medical documentation, proactive monitoring of care transitions, systematic scrutiny of medical records, and patient education in symptom articulation and strategic questioning. This framework was conceptualized based on diagnostic error research demonstrating that process breakdowns most frequently involve the patient-practitioner clinical encounter, particularly history taking (78.9 %), but are also significantly related to referrals (19.5 %), patient-related factors (16.3 %), follow-up and tracking of diagnostic information (14.7 %), and performance and interpretation of diagnostic tests (13.7 %) [70]. The model recognizes these areas as theoretically correctable given sufficient attention and analysis, despite time constraints on clinicians and fragmented medical records [71].

## Looking to the future

Patient engagement and diagnosis are both broad fields. Both, in some fashion, touch every part of healthcare. We have attempted to cover trends in research consciously defined as patient engagement in diagnosis, but there is much excellent research that space does not permit us to go into, including innovative co-design and partnering initiatives originating both in the United States and elsewhere. Additional work is in the research pipeline. Examples include a co-design toolkit on patient engagement methods for diagnostic safety; a natural language processing model for open-ended responses on patient experience surveys; a systematic review of inequities in the diagnostic process for mental disorders; community listening sessions around diagnosis of Alzheimer’s and dementia; and assessing communication in patient encounters using transcripts from ambient scribes [7]. Uses of the PRM roadmapping framework are emerging, through the first published application on prenatal diagnosis in Finland [72]. Project PIVOT, led by patient advocates, also proposes further work on patient perspectives on PRMs [73]. The Center for Patient-Reported Measures of Diagnostic Excellence, launched in 2025, aims to serve as a hub for the development, use, and assessment of diagnostic PRMs [74].

**Figure 4:**
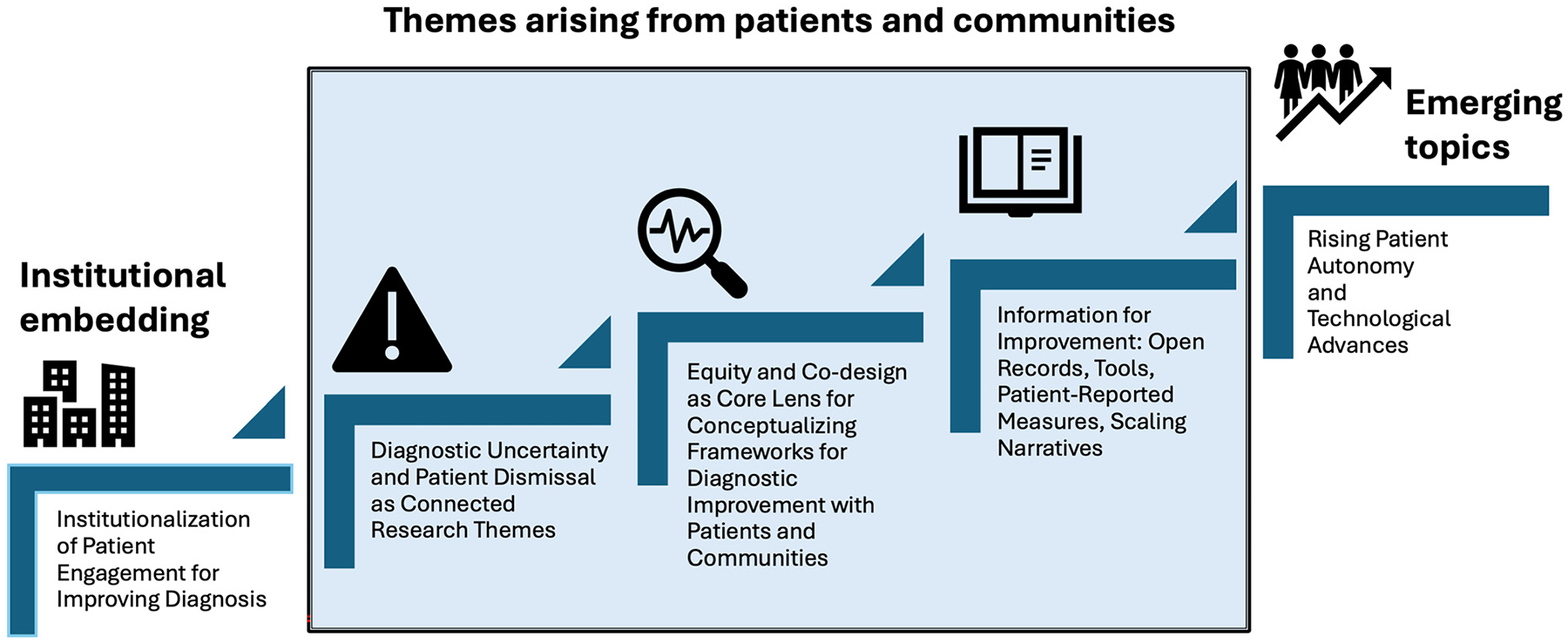
Patient engagement in diagnosis: Major research themes 2015–2025, showing development of institutional support, themes arising from research involving patients and communities, and emerging influences from outside the academic sphere.

The years of research into patient engagement have changed the conversation in diagnosis (Figure 4). The problems of diagnostic inequity and dismissal of patient concerns are now openly discussed and investigated. Patient reporting, once strongly opposed by physicians, now shows promise of becoming policy as patient-reported measures are strengthened. Co-design, another mainstay of patient engagement research, has spread widely in concept if not always in practice. We have the building blocks, at least in theory, for a patient-centered system that extends from communication in the diagnostic encounter through community involvement to national policies defined by measurement. Alongside and intertwined with these developments are the new freedom afforded by advances in technology and the rise in patient autonomy driven by online access to medical information and near real-time access to medical records, a pipe dream only a few years ago. Generative AI has further brought diagnostic healthcare information within the reach of people who may previously have lacked the resources or inclination to seek it out. Surveys indicate that, despite caveats about uneven reliability, Americans are rapidly taking advantage of this new capability.

How these different aspects of patient engagement in diagnosis will ultimately come together is unclear. Of the recommendations in the NASEM report and the “Patient is In” article with which we began this commentary, some but not all have been met, despite prolific and varied research. We have theoretical constructs but do not know their effect in real life. We have access to patient records but have yet to find a way to reach people with basic information about the diagnostic process. Research in general does not take into account the many system constraints, including insurance demands and time compression, that affect the patient experience far more than they did a decade ago.

One reason for these research gaps is the silos that exist even within a single field. More and more publications are read by fewer and fewer people and may have little effect on practice. In addition, competition for funding can inhibit collaboration. In the present climate, as sources of financing dwindle, this may well be exacerbated; yet patient needs are greater than ever. We need a recalibrated master plan, grounded in research findings and public opinion, that takes into account the most pressing needs of patients around diagnosis. In the present rapidly changing environment, it remains to be seen how much of our existing research programs can be implemented in a meaningful way. What is clear is that the role of technology will continue to increase and that patients will not wait for system changes but will continue to find their own way. It is important that researchers work in tandem with patients of all backgrounds to harness their knowledge, experience, and creative energy in proposing solutions for a system that works for all.
